# Supplementary material for: Causal Relationship between Aging and Anorexia Nervosa: A White-Matter-Microstructure-Mediated Mendelian Randomization Analysis
Source: Biomedicines. 2024 Aug 16;12(8):1874. doi: 10.3390/biomedicines12081874 (PMC11351342; doi:10.3390/biomedicines12081874)
Supplement: Supplementary file 1 [file biomedicines-12-01874-s001.zip › Supplementary Figure/Figure S8/Leaveoneout plot of MR analysis for TL to ubm-b-1474.pdf]

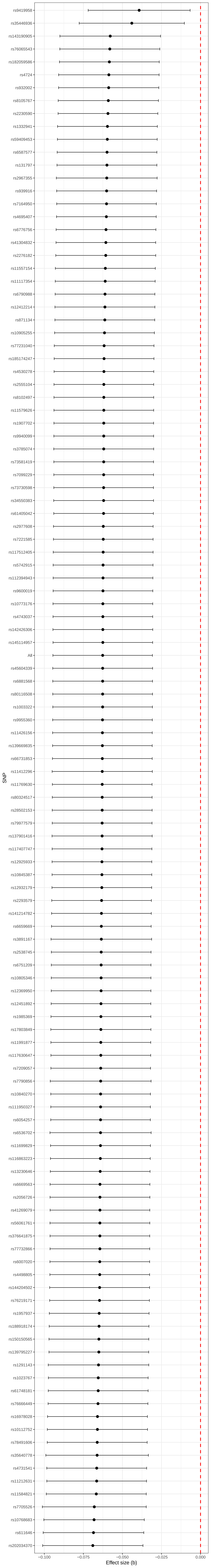
rs9419958
rs35446936
rs143190905
rs76065543
rs182059586
rs4724
rs932002
rs8105767
rs2230590
rs1332941
rs59409453
rs6587577
rs131797
rs2967355
rs939916
rs7164950
rs4695407
rs6776756
rs41304832
rs2276182
rs11557154
rs11117354
rs6790988
rs12412214
rs871134
rs10905255
rs77231040
rs185174247
rs4530278
rs2555104
rs8102497
rs11579626
rs1907702
rs9940099
rs3785074
rs73581419
rs7099229
rs73730598
rs34550383
rs61405042
rs2977608
rs7221585
rs117512405
rs5742915
rs112394943
rs9600019
rs10773176
rs4743037
rs142426306
rs145114957
All
rs45604339
rs6881568
rs80116508
rs1003322
rs9955360
rs11426156
rs139669835
rs66731853
rs11412296
rs11769630
rs80324517
rs28502153
rs79977579
rs137901416
rs117407747
rs12925933
rs10845387
rs12932179
rs2293579
rs141214782
rs6659669
rs3891167
rs2538745
rs6751209
rs10805346
rs12369950
rs12451892
rs1985369
rs17803849
rs11991877
rs117630647
rs7209057
rs7790856
rs10840270
rs111950327
rs6054257
rs6536702
rs11699829
rs116863223
rs13230646
rs6669563
rs2056726
rs41269079
rs56061761
rs376641875
rs77732866
rs6007020
rs4498805
rs144204502
rs76219171
rs1957937
rs188918174
rs150150565
rs139795227
rs1291143
rs1023767
rs61748181
rs76666449
rs16978028
rs10112752
rs78491606
rs35640778
rs4731541
rs11212631
rs11584821
rs7705526
rs10768683
rs611646
rs202034370
SNP
-0.100
-0.075
-0.050
-0.025
0.000
Effect size (b)
